# Comparative analyses of immune cells and alpha-smooth muscle actin-positive cells under the immunological microenvironment between with and without dense fibrosis in primary central nervous system lymphoma

**DOI:** 10.1007/s10014-024-00488-7

**Published:** 2024-08-26

**Authors:** Jun Takei, Miku Maeda, Nei Fukasawa, Masaharu Kawashima, Misayo Miyake, Kyoichi Tomoto, Shohei Nawate, Akihiko Teshigawara, Tomoya Suzuki, Yohei Yamamoto, Hiroyasu Nagashima, Ryosuke Mori, Ryoko Fukushima, Satoshi Matsushima, Hiroyoshi Kino, Ai Muroi, Takao Tsurubuchi, Noriaki Sakamoto, Kaichi Nishiwaki, Shingo Yano, Yuzuru Hasegawa, Yuichi Murayama, Yasuharu Akasaki, Masayuki Shimoda, Eiichi Ishikawa, Toshihide Tanaka

**Affiliations:** 1 Department of Neurosurgery, The Jikei University Katsushika Medical Center, 6-41-2 Aoto, Katsushika-ku, Tokyo, 125-8506 Japan; 2 Department of Neurosurgery, The Jikei University School of Medicine, 3-25-8 Nishi-Shimbashi, Minato-Ku, Tokyo, 105-8461 Japan; 3 Department of Pathology, The Jikei University School of Medicine, 3-25-8 Nishi-Shimbashi, Minato-ku, Tokyo, 105-8461 Japan; 4 Division of Clinical Oncology and Hematology, The Jikei University School of Medicine, 3-25-8 Nishi-Shimbashi, Minato-ku, Tokyo, 105-8461 Japan; 5 Department of Pathology, The Jikei University Kashiwa Hospital, 163-1 Kashiwa-shita, Kashiwa, Chiba, 277-8567 Japan; 6 Department of Neurosurgery, The Jikei University Kashiwa Hospital, 163-1 Kashiwa-shita, Kashiwa, Chiba, 277-8567 Japan; 7 Department of Neurosurgery, The Jikei University Daisan Hospital, 4-11-1 Izumi-honcho, Komae-shi, Tokyo, 201-8601 Japan; 8 Division of Clinical Oncology and Hematology, The Jikei University Kashiwa Hospital, 163-1 Kashiwa-shita, Kashiwa, Chiba, 277-8567 Japan; 9 Department of Radiology, The Jikei University School of Medicine, 3-25-8 Nishi-Shimbashi, Minato-ku, Tokyo, 105-8461 Japan; 10 Department of Neurosurgery, Institute of Medicine, University of Tsukuba, 1-1-1 Tennodai, Tsukuba, Ibaraki, 305-8575 Japan; 11 Department of Clinical Pathology, Institute of Medicine, University of Tsukuba, 1-1-1 Tennodai, Tsukuba, Ibaraki, 305-8575 Japan

**Keywords:** Primary central nervous system lymphoma, Dense fibrosis, Immunological tumor microenvironment, Alpha-smooth muscle actin-positive cells, Pathology

## Abstract

Histopathologic examinations of primary central nervous system lymphoma (PCNSL) reveal concentric accumulation of lymphocytes in the perivascular area with fibrosis. However, the nature of this fibrosis in “stiff” PCNSL remains unclear. We have encountered some PCNSLs with hard masses as surgical findings. This study investigated the dense fibrous status and tumor microenvironment of PCNSLs with or without stiffness. We evaluated by silver-impregnation nine PCNSLs with stiffness and 26 PCNSLs without stiffness. Six of the nine stiff PCNSLs showed pathological features of prominent fibrosis characterized by aggregation of reticulin fibers, and collagen accumulations. Alpha-smooth muscle actin (αSMA)-positive spindle cells as a cancer-associated fibroblast, the populations of T lymphocytes, and macrophages were compared between fibrous and control PCNSLs. Fibrous PCNSLs included abundant αSMA-positive cells in both intra- and extra-tumor environments (5/6, 87% and 3/6, 50%, respectively). Conversely, only one out of the seven control PCNSL contained αSMA-positive cells in the intra-tumoral area. Furthermore, the presence of extra-tumoral αSMA-positive cells was associated with infiltration of T lymphocytes and macrophages. In conclusion, recognizing the presence of dense fibrosis in PCNSL can provide insights into the tumor microenvironment. These results may help stratify patients with PCNSL and improve immunotherapies for these patients.

**Supplementary Information:**

The online version contains supplementary material available at 10.1007/s10014-024-00488-7.

## Introduction

Primary central nervous system (CNS) lymphoma (PCNSL) is a type of malignant brain tumor, accounting for 2–4% of all intracranial tumors [1, 2]. The most common form of PCNSL is diffuse large B-cell lymphoma of the CNS (CNS-DLBCL).

Histologically, PCNSL usually consists of highly cellular, diffusely growing mature late germinal center exit B cells with CD20 and CD79. Necrosis, perivascular infiltration pattern is frequent. Tumor cells are intermingled with reactive inflammatory T lymphocytes, including CD3 and CD8 positive, reactive glial fibrillary acidic protein (GFAP)-positive astrocytes, and CD68 positive macrophages [3].

While treatments such as high-dose methotrexate are available, 15–25% of patients do not respond to chemotherapy, and 25–50% experience relapse after the initial response [4]. To improve patient management and ensure prompt treatment, general recommendations have been published [4]. Gadolinium-enhanced magnetic resonance imaging (MRI) is the standard imaging modality used to confirm suspected PCNSL, define the extent of CNS tumor, monitor the tumor during follow-up, and confirm tumor recurrence [5].

When PCNSL is suspected, stereotactic biopsy via burr hole surgery is recommended [6]. PCNSL occurs as single or multiple masses in the brain parenchyma., most frequently seated deep in the cerebral hemisphere adjacent to the ventricular system. Macroscopically, the tumors appear friable, granular, hemorrhagic, and greyish-tan or yellow with areas of necrosis. A small proportion of tumors are extremely firm or calcified. However, we experienced there were some cases where the forceps slipped during a biopsy, and it was necessary to be cautious with certain cases of CNS lymphomas. Upon considering the reasons, we realized that one cause is the presence of stiffness of tumors. The importance of this perspective is that when the tumor is extremely stiff, craniotomy might be necessary to remove the tumor safely instead of a stereotactic biopsy.

To date, clinical and basic research on the stiffness sometimes seen in PCNSL has not been conducted. Solid tumors are typically characterized by a build-up of extracellular matrix (ECM), along with remodeling and cross-linking. This process leads to fibrosis that stiffens the stroma and promotes malignancy [7]. The aggressiveness of tumor growth and clinical outcomes for patients correlate with the degree of tissue fibrosis and stromal stiffness via crosstalk among tumor cells, immune cells, and fibroblasts, suggesting that tissue fibrosis might provide another target in cancer therapy [7].

In addition, histopathological examination of PCNSL usually reveals a prominent accumulation of reticulin fibers, concentrated in the perivascular space [8, 9]. However, few studies have explored the correlation between the degree of fibrosis and the accumulation of reticulin fibers in PCNSL. Research examining the utility of MRI perfusion imaging features for differentiating between PCNSL and glioblastoma (GB) found that rich formation of reticular fibers was a typical feature of PCNSL that might slow the diffusion of contrast agent molecules into the interstitium, thus altering time signal intensity curves for PCNSL [10]. Thus, depending on the degree of fibrosis, PCNSL might show different biological characteristics due to the particular tumor microenvironment (TME) in the interstitium.

We have encountered some cases with PCNSL showing stiffness as a surgical finding. While investigating the cause of this stiffness, we noticed a gap in the research regarding fibrosis in PCNSL. This study aimed to explore the characteristics of PCNSL exhibiting marked fibrosis.

## Methods

### Patients

The study protocol was approved by the institutional review boards of The Jikei University School of Medicine [permission number: 35–181(11,810)] and University of Tsukuba [permission number: R05-124]. We implemented an opt-out method that was approved by the institutional review board to acquire consent for this retrospective study. Our institutional homepage has a notice that provides the necessary information.

This study included two different cohorts. First, we extracted data on ten tumors with documented stiffness according to the surgical records of patients with PCNSL. These records were included based on neurosurgeon descriptions clearly mentioning tumor stiffness. This cohort consisted of cases from the Departments of Neurosurgery at The Jikei University Hospital, The Jikei University Katsushika Medical Center, The Jikei University Kashiwa Hospital, and University of Tsukuba treated between January 2015 and June 2023. Next, 43 tumors were extracted from surgical records of consecutive PCNSL cases without “descriptions of stiffness”. Patient data, such as age, sex, surgical procedure, treatment, and overall survival were obtained from electronic charts and surgical records. Cases with secondary CNS lymphoma, histology other than DLBCL, immune human immunodeficiency virus (HIV)-related lymphoma, preoperative steroid administration, and lack of tumor sample for analysis were excluded from the present study.

### Histopathology and evaluation of fibrous status

**Fig. 1:**
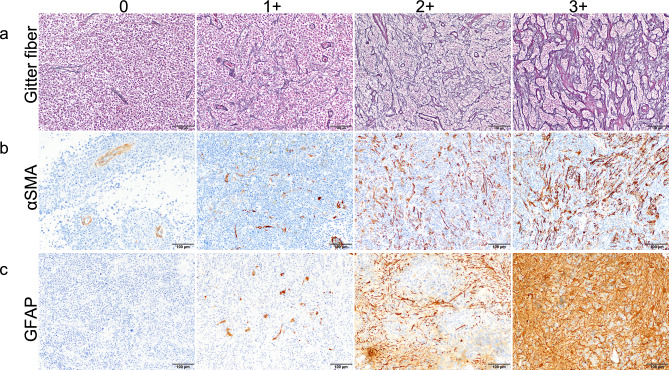
Definitions for histopathological categorization. **a** Reticular fiber expression was scored as the degree of reticular fiber cross-linking and aggregation. **b**, **c** Both αSMA-positive spindle cells and GFAP-positive cells were classified into four levels according to density. Original magnifications: **a** × 100; **b**, **c** × 200. Scale bars: **a** 200 μm; **b**, **c** 100 μm

All surgical specimens were fixed in 10% neutral-buffered formalin solution (Muto Pure Chemicals, Tokyo, Japan) or 10% formalin (Mitsubishi Gas Chemical, Tokyo, Japan), processed and embedded in paraffin, creating formalin-fixed paraffin-embedded (FFPE) sections. FFPE tissues were thinned to 4 μm for staining with hematoxylin and eosin (HE) and Masson’s trichrome (MT). All selected PCNSL cases were reviewed by two pathologists (MM and NF) and classified into germinal center B (GCB) or non-GCB subtypes according to expression of CD10, Bcl6, and MUM1 determined by immunohistochemistry [11]. FFPE tissues were also thinned to 7.5 μm for silver-impregnation using Watanabe’s method, to identify reticular fiber as black structure and collagen formation as red structure. We scored reticular fibers from 0 to + 3 according to the following criteria (excluding the perivascular reticulin network): 0, no cross-linking at all; + 1, slight cross-linking; + 2, moderate cross-linking; and + 3, prominent aggregation with collagenous stroma deposition (Fig. 1a). Cases scoring + 3 for reticular fibrosis were taken as the fibrosis group (FG). All other cases were allocated to the control group (CG).

### Immunohistochemistry and evaluation

Immunohistochemical analysis using antibodies for CD20, CD3, CD4, CD8, programmed cell death 1 (PD-1), forkhead box P3 (Foxp3), CD68, CD163, α-smooth muscle actin (αSMA), and glial fibrillary acidic protein (GFAP) were performed on 4-μm FFPE sections. Details of each antibody are presented in Supplemental Table 1. Automated immunohistochemistry (IHC) with the CD20, CD3, CD4, CD8, PD1, CD68, CD163, αSMA, and GFAP were performed in a BenchMark-ULTRA autostainer or Discovery-ultra autostainer (Ventana Medical Systems, Tucson, AZ, USA; division of Roche Diagnostics, Basel, Switzerland). In brief, slides were deparaffinized using EZ prep (catalog no. 950–102; Roche) followed by antigen retrieval (cell conditioning solution [CC1], catalog no. 951–124; Roche) at the necessary temperature and time presented in Supplemental Table 1. After retrieval, slides were blocked for peroxidase for 4 min. IHC was performed with incubation for each antibody at the required temperature and time. An Ultra-View DAB Universal kit (catalog no. 760–500; Roche) was used according to the recommendations from the manufacturer for visualization of the bound primary antibody. Slides were then counterstained with hematoxylin II (catalog no. 790–2208; Roche) for 8 min followed by bluing reagent (catalog no. 760–2037; Roche) for 4 min, prior to dehydration in a graded alcohol series. For IHC with Foxp3, slides were deparaffinized followed by antigen retrieval in citrate buffer (pH 6.0) in an autoclave. Slides were incubated with anti-Foxp3 antibody (Supplemental Table 1) at 4 °C overnight.

**Table 1:** Baseline characteristics of patients with PCNSL

	Fibrosis group	Control group (non-fibrous group)	Total	*p*-value
Numbers	6	7	13	
Sex				*p* = 1.000*
Woman	4	4	8	
Man	2	3	5	
Age –year Median IQR	69 65–79	66 54–78	66 62–78	*p* = 0.830†
GCB subtype				*p* = 1.000*
GCB	2	2	4	
Non-GCB	4	5	9	
Surgical procedure				*p* = 0.324*
Biopsy	1	4	5	
Open biopsy	2	2	4	
Partial removal	3	1	4	
Treatment				*p* = 0.767*
MTX-based	3	3	6	
R-MPV	2	4	6	
Rituximab	1	0	1	
Overall survival-month				
Median	59	undefined	59	*p* = 0.992††

To assess CD3, CD4, CD8, Foxp3, PD-1, CD68, and CD163 expressions, stained sections were surveyed under a low-power field (× 40). In each specimen, three hot spots were selected in the tumor center and three in the tumor margins, making a total of six spots. Positive cells in these areas were counted in three high-power fields (× 400, total 0.71 mm^2^) by one neurosurgeon and one pathologist (JT and MM), as previously described [12, 13]. For quantitative evaluation of αSMA and GFAP, stained sections were screened in a low-power field (× 40) and a middle-power field (× 200) showing the densest spots in the tumor center, then margins were assessed in 4 categories (0 to 3 + , as shown in Fig. 1b, c) by two pathologists (MM and NF). Expression of αSMA was qualified using the following criteria (Fig. 1b): 0, αSMA positivity detected only in the vessel wall; + 1, αSMA-positive spindle cells scattered in small numbers outside the vessel wall; + 2, αSMA-positive spindle cells moderately distributed with partial cross-linking; and + 3, αSMA-positive spindle cells distributed at high density. We used αSMA as a cancer-associated fibroblast (CAF) marker [14, 15] and categorized the expression of αSMA focusing exclusively on spindle-shaped cells as shown in supplemental Fig. 1. GFAP evaluation (Fig. 1c) used the following criteria: 0, no or few GFAP-positive cells; + 1, a few GFAP-positive gemistocytic astrocytes; + 2, moderate number of gemistocytic or fibrillary astrocytes; + 3, a large number of gemistocytic or fibrillary astrocytes with dense distribution. If these findings were only very localized in the specimen, cases were classified into a lower category. We used GFAP to distinguish gliosis. Cells were counted using Fiji software (version 2.0.0-re-69/1.52p) [16]. All photos were captured from the identical microscopic field. This ensured that similar locations were chosen across the specimens for CD3, CD4, CD8, Foxp3, PD-1, CD68, CD163, αSMA, and GFAP.

### Statistics

Continuous data are expressed as the median and interquartile range (IQR), and categorical data as numbers and percentages. To compare characteristics between the two groups, the Mann–Whitney U test (MWU) and paired-t-tests were used, as appropriate. Correlation analysis was used to measure the strength of the linear relationship between two variables. To compare the survival time between the two groups, a log-rank test was used. Cases with missing data were omitted, and the remaining available data were analyzed. Statistical analyses were performed using STATA version 18 (StataCorp LLC, College Station, TX, USA) or GraphPad Prism version 10 (GraphPad Software, Boston, MA, USA). All p-values were two-sided, with values of p < 0.05 considered significant.

## Results

**Fig. 2:**
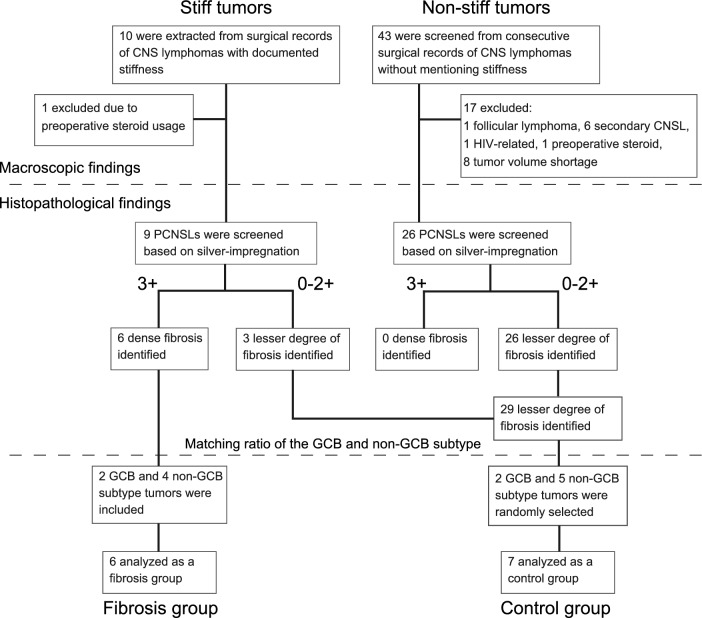
Flowchart of patients included in the present study

A flow chart showing the algorithm for registration is provided in Fig. 2. Among the ten tumors with documented stiffness in surgical records, one was excluded due to preoperative steroid usage. In the consecutive 43 tumors without documented stiffness, 17 tumors were excluded because of preoperative steroid usage (*n* = 1), treatment history of systemic follicular lymphoma (*n* = 1), secondary CNS lymphoma (*n* = 6), HIV-related lymphoma, and insufficient tumor sample (*n* = 8). The remaining nine tumors with stiffness and 26 tumors without stiffness were screened by histopathological analyses of HE staining and silver-impregnation. Among the nine stiff tumors, six tumors showed dense fibrosis confirmed by silver staining and were defined as the fibrosis group (FG). The remaining three stiff tumors and the 26 non-stiff tumors showed a lesser degree of fibrosis. To compare the FG and these 29 tumors with a lesser degree of fibrosis, we matched GCB and non-GCB subtypes.

The FG included two GCB subtype tumors and four non-GCB subtype tumors. Among other 29 tumors showing a lesser degree of fibrosis, we randomly selected 7 samples for the control group (CG), including 2 GCB subtype tumors and 5 non-GCB subtype tumors, by matching the GCB and non-GCB subtype ratio. This random selection was performed before the subsequent immunohistochemistry analysis. Demographic data for all patients are shown in Table 1. No significant differences between groups were identified in terms of age, sex, surgical procedure, treatment, and survival time. Details of each patient are presented in Supplemental Table 2.

### Pathological findings of fibrous PCNSL from a representative case (Case 2, supplementary Table 2)

**Fig. 3:**
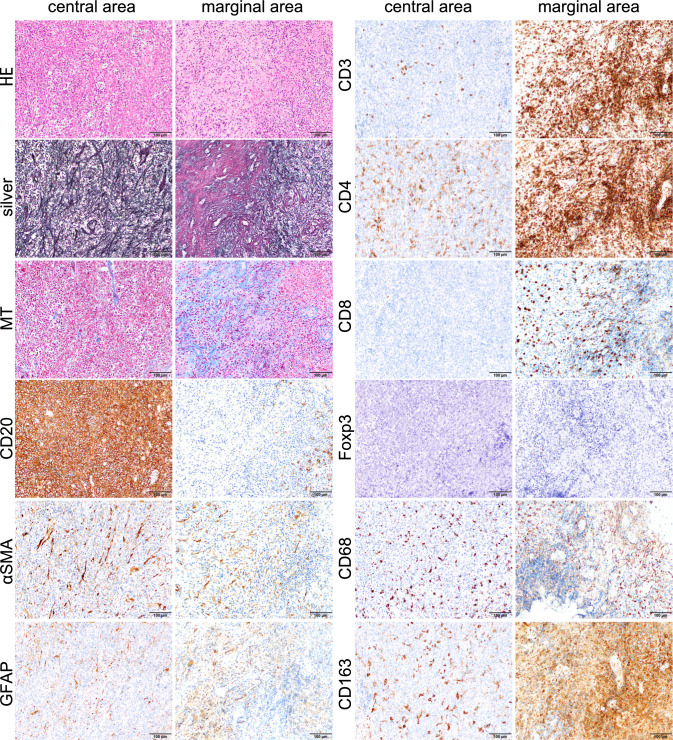
Histopathological findings of fibrosis in the representative case (Case 2, Supplementary Table 2). The histopathological finding of the representative case of fibrosis PCNSL. Hematoxylin and eosin staining of the specimen shows the central and marginal areas of the tumor. Silver-impregnation showed increased reticular fibers bridging blood vessels in the central area of the tumor, and dense fibrosis and vascular aggregation with collagen deposition stained red in the marginal area. Masson’s trichrome showed collagen deposition in the marginal area. CD20-positive cells are sparse in the marginal area. Many αSMA-positive spindle cells exist both in the central and marginal areas. GFAP-positive glial cells are sparse in both areas. CD3-, CD4, CD8, CD68, and CD163-positive lymphocytes are abundant in the marginal areas of the tumor. Foxp3-positive cells are few in both areas. Original magnification: × 200; scale bar: 100 μm

Histopathological examination of the tumor identified large B-cell lymphoma characterized by the infiltration and proliferation of CD20-positive centroblast-like atypical cells in a diffuse and high cell density (Fig. 3).

Silver-impregnation showed increased reticular fibers bridging blood vessels in the central area of the tumor, and dense fibrosis and vascular aggregation with collagen deposition stained red by silver-impregnation were observed in the marginal area (Fig. 3). Compared to the central area, the marginal area had more small lymphocytes, especially CD3-positive T cells, and CD4-positive cells were more predominant than CD8-positive cells. Additionally, the marginal area included more CD68- and CD163-positive cells than the central area. In both areas, there was an increase in fibroblast-like spindle-shaped cells that were distributed individually and were positive for αSMA. GFAP-positive astrocytes were patchily observed in both area (Fig. 3). (Scale bar: 100 μm; central and marginal areas were taken from the same area).

### Fibrosis and immunohistochemical characteristics

**Fig. 4:**
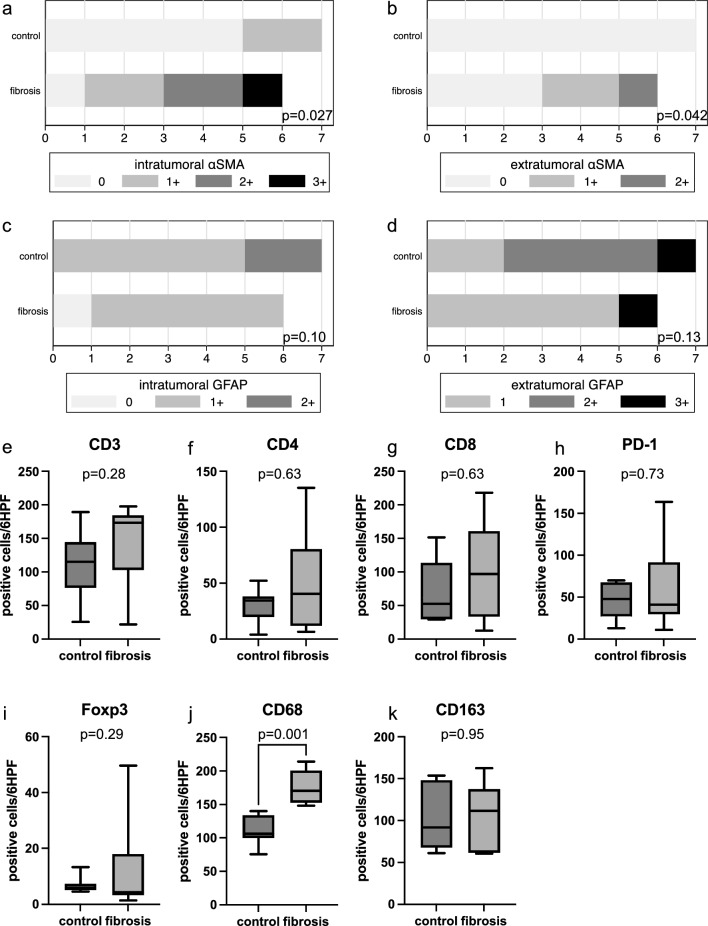
Immunohistochemical analysis of tumor αSMA-positive, GFAP-positive, and immune cells. Immunohistochemical analysis of surgical specimens was performed on 13 samples of PCNSL. **a–d** Expression of αSMA-positive, GFAP-positive cells in tumor specimens among the central and marginal areas. **e–k** CD3-, CD4-, CD8-, PD-1-, Foxp3-, CD68, and CD163-positive cells in the control and fibrous PCNSL groups (*n* = 13)

The FG showed intra-tumoral αSMA-positive cells in 5 of the 6 cases, significantly more than the 2 of 7 cases in the CG (*p* = 0.027, MWU) (Fig. 4a). In addition, extra-tumoral αSMA-positive cells were seen in 3 of 6 FG cases but no CG cases (*p* = 0.042, MWU) (Fig. 4b). More GFAP-positive cells were present in the CG at the tumor margins than in the tumor center, although no significant difference from the FG were identified (Fig. 4c, d, MWU). In comparing the FG and CG, average numbers of CD3-, CD4-, CD8-, PD-1-, and Foxp3-positive cells tended to be higher in the FG. However, no significant differences were identified between groups (Fig. 4e–i, MWU). CD68-positive cells were significantly higher in FG, while CD163-positive cells showed no difference (Fig. 4j, k *p*= 0.001 and p-0.95, respectively, MWU).

### Association between immune cells and fibrotic status

**Fig. 5:**
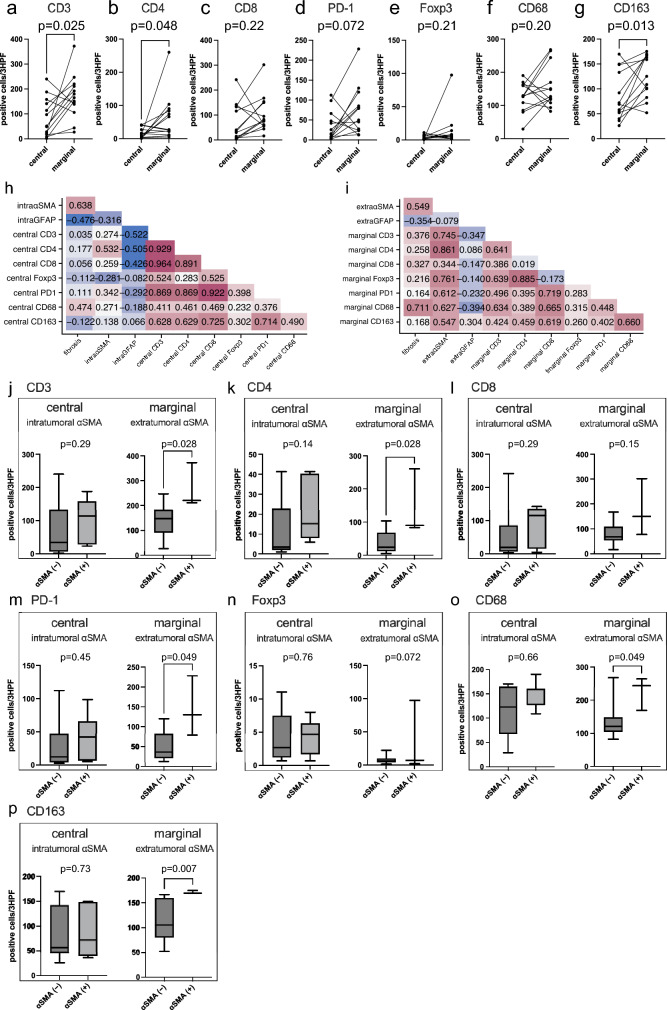
Correlation analysis of the number of immune cells depending on area and tumor fibrosis. **a–g** Comparison between central and marginal areas of CD3-, CD4-, CD8-, PD-1-, Foxp3-, CD68, and CD163-positive immune cells (*n* = 13). **h** Correlation matrix of fibrosis and the tumor microenvironment of the central area. **i** Correlation matrix of fibrosis and the tumor microenvironment of the marginal area. **j–p** Comparison of numbers of CD3-, CD4-, CD8-, PD-1-, Foxp3-, CD68, and CD163-positive immune cells in negative (*n* = 6) and positive (*n* = 7) αSMA scores in the central area of the tumor, as well as negative (*n* = 10) and positive (*n* = 3) αSMA scores in the marginal areas

We conducted a comparison of immune cells based on location. Marginal sites included significantly higher concentrations of CD3-, CD4-, and CD163-positive cells than central sites (Fig. 5a, b, g, paired t-tests) and a tendency toward more abundant CD8-, PD-1-, Foxp3, and CD68-positive cells, although the difference was not significant (Fig. 5c–f, paired t-tests). Next, correlation analysis based on tumor area revealed a positive correlation between fibrosis and intra- and extra-tumoral αSMA expression (correlation coefficients 0.638 and 0.549, respectively), but a negative correlation with intra- and extra-tumoral GFAP expression (correlation coefficients -0.476 and -0.354, respectively) (Fig. 5h, i). In addition, extra-tumoral αSMA expression showed a strong positive correlation with infiltration of CD3-, CD4-, Foxp3-, PD1-, CD68-, and CD163-positive cells (Fig. 5i). Conversely, intra-tumoral GFAP expression correlated negatively with infiltration of CD3-, CD4-, and CD8-positive cells (Fig. 5h). Finally, we investigated whether the presence of αSMA-positive cells affected the tumor-infiltrating lymphocytes (TILs). We defined tumors with an intra- or extra-tumoral αSMA score of 0 as negative. Extra-tumoral αSMA-positive tumors included significantly more CD3-, CD4-, PD-1-, CD68- and CD163-positive cells than negative tumors (Fig. 5j–p, MWU).

## Discussion

This study found the presence of dense fibrosis only in tumors showing extreme stiffness when obtained via biopsy. Pathological findings for these tumors showed infiltration and proliferation of atypical centroblast-like cells in diffuse and high cell densities, along with a high degree of fibrosis and collagen accumulation in tumor interstitial tissues. Based on pathological findings including immunohistochemical staining for CD20, all biopsied tumors in the present study showing stiffness from dense fibrosis were diagnosed as PCNSL. In this study, we demonstrate the existence of a subset of stiff PCNSL with dense fibrosis. While stereotactic biopsy is recommended when PCNSL is suspected, [6] our findings support the importance of surgeon’s sense of the tumor’s stiffness during surgery, and suggest considering switching to craniotomy when necessary.

Next, we found that the degree of tumor fibrosis causing stiffness might induce reciprocal interplay between tumor cells and stromal cells containing fibroblasts [7]. FG tumors showed prominent fibrosis that obscured the perivascular space, as if vessels were adjacent to each other amidst the diffuse tumor cell infiltration. This contrasted with the concentric accumulation of reticular fibers that encapsulated tumor cells extending along the perivascular space, which is a typical feature of CNS-DLBCL. In addition, the stiff tumor showed a proliferation of αSMA-positive spindle cells but tended to be fewer GFAP-positive glial cells than control tumors (Fig. 4a). This suggested that stiffness in PCNSL may primarily result from cancer-associated fibroblasts (CAFs) rather than reactive astrocytes. To detect CAFs, αSMA used as a general marker for vascular, muscular cells, and pericytes is a well-established marker for this perspective [14, 15] αSMA-positive CAFs can cause ECM remodeling by secreting collagen I, thereby increasing the stiffness of the tumor [14].

Uncertainty remains regarding whether CAFs exist in CNS tumors, but the advent of new technologies such as single-cell RNA sequencing is giving rise to many insights in this field. Recent evidence suggests that fibroblasts exist in perivascular spaces, meninges, and the choroid plexus of the brain and spinal cord [17–19]. CNS fibroblasts contribute to the development and pathology of the CNS, including inflammatory diseases, infections, and injuries [20]. In terms of CNS tumors, some studies have identified tumor-associated stromal cells expressing CAF markers in GB and have discovered protumoral effects of αSMA- or platelet-derived growth factor receptor-beta-positive fibroblasts [21]. Such fibroblasts are defined as CAFs that increase the growth potential of tumor cells, based on the gene expression profiles in GB [21]. Other studies have noted the importance of marrow-derived precursors instead of local fibroblasts, giving rise to CAFs in other types of tumors [22, 23].

CAFs play a pivotal role in the TME and show diverse functions, including ECM deposition and remodeling, extensive reciprocal signaling interactions with cancer cells, and crosstalk with infiltrating leukocytes [24]. CAFs are also known to impact cancer development by engineering tumor cell tension and modulating immune responses [7]. αSMA-positive CAFs within the tumor microenvironment increase tumor-associated macrophages (TAMs), particularly contributing to the increase of M2 macrophages and further support an immunosuppressive TME by reducing T cell activity [14]. CAFs may be present in GB. Based on such findings, attention must shift to determining whether CAFs are present in PCNSL. To the best of our knowledge, this represents the first report to show that dense fibrosis comprising reticulin fibers in stiff PCNSL contained CAFs.

Another important finding of this study was that CAFs were associated with marginal TILs. We found that PCNSLs showing extra-tumoral CAFs also displayed significantly higher levels of CD3-, CD4-, PD-1-, CD68, and CD163-positive cells compared with CAF-negative PCNSL, but only in the marginal area of tumors. These results are consistent with earlier studies. Jain et al. showed that CAFs increased the percentage of M2 macrophages in GBM6 neuro-sphere-derived tumors in vivo [21]. Further, CAFs increase ECM secretion, creating a physical barrier to prevent immune cell infiltration and migration within the tumor, based on the secretion of chemokine CXCL12, which is known to promote angiogenesis and tumor growth. This in turn enhances T-cell chemotaxis toward CAFs, both in human pancreatic cancer specimens and mouse models [25, 26]. These studies may support previous observations that T cells in prominent fibrous regions were conspicuously separated from their cancer cell targets. On the other hand, CAFs reduce the proliferation of both CD4- and CD8-positive T cells in vitro [27]. By secreting immunosuppressive factors like CXCL12, TGF-β, vascular endothelial growth factor, and nitric oxide [25, 27]. CAFs suppress anti-tumor immune responses via the modulation of immune cell activity through the induction and inhibition of various cytokines. Overall, CAFs play a significant role in cancer progression through induction of an immunosuppressive TME [28]. Our findings suggest that PCNSLs showing dense fibrosis create a different TME, especially in terms of abundant TAM infiltrations, to that of non-fibrous PCNSLs, which might raise issues of immunological “hot” or “cold” tumors. Previous research showed that a higher presence of TAMs in PCNSL is associated with poor prognosis [29]. Furthermore, immune checkpoint inhibitors may be effective in treating PCNSL [30–32]. These results suggest that immunotherapy for PCNSL represents a novel therapeutic strategy. Although this study did not show a difference in survival time between fibrous and non-fibrous PCNSLs, fibrous PCNSLs had higher numbers of TAMs and PD-1-positive cells compared with non-fibrous PCNSLs. This suggests that fibrous PCNSLs might respond well to immunotherapy that targets TAMs and immune checkpoints.

This study had a few limitations that need to be considered. First, sample selection was limited due to the retrospective design of the investigation, which could have led to selection bias. Second, tumor stiffness was assessed subjectively by the surgeon and was not able to be objectively measured. However, the finding that dense fibrosis was only observed in tumors described as “stiff” tumors suggests that a subset of PCNSLs differs from the “non-stiff” PCNSLs. Third, CAFs in this study were defined as αSMA-positive, spindle-shaped cells. However, different subtypes of CAFs show various phenotypes, each with different functions in the TME [28]. Thus, clinically relevant organoid models of PCNSL would need to track details that have yet to be established. Fourth, this study might underestimate the impact of secondary fibrosis due to necrosis, reactive changes, or inflammatory changes. This is because this study evaluated fragmented tumor tissue obtained from biopsies. Fifth, the causal relationship between fibrosis and CAF proliferation has not been proven. Despite these limitations, we believe that CAF infiltration in the group of fibrous PCNSL might have exerted a robust impact on the immunological TME. Clarification of those impacts may well prove useful in understanding the pathogenesis of these tumors, the development of novel therapeutic approaches, and the prediction of clinical outcomes.

## Conclusions

We found that a proportion of PCNSL showed pathological features of dense fibrosis characterized by tumor stiffness with increasing reticular fibers and collagen accumulation. Fibrous PCNSLs mostly have αSMA-positive spindle cells in the interstitial tissues of both intra- and extra-tumoral regions. In addition, the presence of extra-tumoral αSMA-positive spindle cells is associated with infiltration of lymphocytes and macrophages. Both αSMA-positive spindle cells and immune cells might contribute to dense fibrosis in PCNSL.

## Supplementary Information

Below is the link to the electronic supplementary material.

Supplementary file1 (DOCX 30 kb)

Supplementary file2 (XLSX 10 kb)

## Data Availability

The datasets generated during and/or analyzed during the current study are available from the corresponding author on reasonable request.
