# Supplementary material for: Comparative analyses of immune cells and alpha-smooth muscle actin-positive cells under the immunological microenvironment between with and without dense fibrosis in primary central nervous system lymphoma
Source: Brain Tumor Pathol. 2024 Aug 26;41(3-4):97–108. doi: 10.1007/s10014-024-00488-7 (PMC11499374; doi:10.1007/s10014-024-00488-7)
Supplement: Supplementary file 1 — Supplementary file1 (DOCX 30 kb) [file 10014_2024_488_MOESM1_ESM.docx]

| Supplementary table 2 | | | |  |  |  |  |  |  |  |  |  |  |  | |
| --- | --- | --- | --- | --- | --- | --- | --- | --- | --- | --- | --- | --- | --- | --- | --- |
| Case | Age/  Sex | Group | GCB subtype | Treatment | Overall survival-months | Event | Renti-culin score | Intra  tumoral αSMA | Extra  tumoral αSMA | Intra  tumoral GFAP | Extra  tumoral GFAP | central CD3 (/3HPF) | marginal CD3 (/3HPF) | total CD3 (/6HPF) | |
| 1 | 66/W | FG | non-GCB | MTX-based | 2.6 | dead | 3 | 2 | 0 | 0 | 1 | 188.0 | 144.7 | 166.3 | |
| 2 | 79/W | FG | non-GCB | R-MPV | 30 | censored | 3 | 2 | 2 | 1 | 1 | 23.0 | 372.0 | 197.5 | |
| 3 | 72/W | FG | non-GCB | Rituximab | 6 | dead | 3 | 1 | 1 | 1 | 3 | 48.7 | 210.7 | 129.7 | |
| 4 | 65/W | FG | GCB | MTX-based | 59 | dead | 3 | 3 | 1 | 1 | 1 | 141.0 | 220.0 | 180.5 | |
| 5 | 32/M | FG | GCB | R-MPV | 43 | censored | 3 | 1 | 0 | 1 | 1 | 114.0 | 247.0 | 180.5 | |
| 6 | 79/M | FG | non-GCB | MTX-based | 14 | censored | 3 | 0 | 0 | 1 | 1 | 17.0 | 27.0 | 22.0 | |
|  |  |  |  |  |  |  |  |  |  |  |  |  |  |  | |
| 7 | 66/M | CG | GCB | R-MPV | 19.6 | censored | 1 | 0 | 0 | 1 | 2 | 240.3 | 138.7 | 189.5 | |
| 8 | 62/W | CG | non-GCB | R-MPV | 19.9 | censored | 1 | 0 | 0 | 1 | 2 | 1.7 | 151.0 | 76.3 | |
| 9 | 54/M | CG | GCB | R-MPV | 8.4 | dead | 2 | 0 | 0 | 1 | 1 | 51.0 | 179.7 | 115.3 | |
| 10 | 52/W | CG | non-GCB | R-MPV | 37.8 | censored | 1 | 0 | 0 | 2 | 3 | 7.7 | 43.7 | 25.7 | |
| 11 | 74/W | CG | non-GCB | MTX-based | 1 | censored | 0 | 0 | 0 | 1 | 1 | 97.0 | 192.0 | 144.5 | |
| 12 | 79/W | CG | non-GCB | MTX-based | 4 | censored | 0 | 1 | 0 | 2 | 2 | 28.0 | 165.3 | 96.7 | |
| 13 | 78/M | CG | non-GCB | MTX-based | 3 | dead | 1 | 1 | 0 | 1 | 2 | 158.0 | 106.0 | 132.0 |  |
| CG: control group, FG: fibrous group, Foxp3: forkhead box P3, GCB: germinal center B, GFAP: glial fibrillary acidic protein, HPF: high power field, M: men, MTX: methotrexate, PD-1: programmed cell death protein-1, R-MPV: rituximab, methotrexate, procarbazine, vincristine, αSMA: smooth muscle alpha-actine, W: women | | | | | | | | | | | | | | |  |

| Supplementary table 2 continued | | | |  |  |  |  |  |  |  |  |  |  |  |  |
| --- | --- | --- | --- | --- | --- | --- | --- | --- | --- | --- | --- | --- | --- | --- | --- |
| Case | central CD4 (/3HPF) | marginal CD4 (/3HPF) | total CD4 (/6HPF) | central CD8 (/3HPF) | marginal CD8 (/3HPF) | total CD8 (/6HPF) | central PD-1 (/3HPF) | marginal PD-1 (/3HPF) | total PD-1 (/6HPF) | central Foxp3 (/3HPF) | marginal Foxp3 (/3HPF) | total Foxp3 (/6HPF) | central CD68 (/3HPF) | marginal CD68 (/3HPF) | total CD68 (/3HPF) |
| 1 | 40.3 | 25.3 | 32.8 | 142.3 | 56.3 | 99.3 | 47.7 | 24.3 | 36.0 | 2.7 | 6.0 | 4.3 | 190 | 118 | 154 |
| 2 | 10.3 | 260.3 | 135.3 | 4.0 | 77.3 | 40.7 | 5.0 | 130.0 | 67.5 | 1.7 | 97.7 | 49.7 | 127 | 244 | 185.5 |
| 3 | 6.0 | 90.3 | 48.2 | 40.0 | 150.3 | 95.2 | 6.7 | 79.0 | 42.8 | 8.0 | 7.0 | 7.5 | 126.7 | 169.7 | 148.2 |
| 4 | 41.3 | 83.7 | 62.5 | 135.0 | 301.7 | 218.3 | 98.7 | 228.3 | 163.5 | 0.7 | 2.0 | 1.3 | 128 | 264.7 | 196.3 |
| 5 | 15.3 | 11.7 | 13.5 | 115.7 | 167.7 | 141.7 | 66.3 | 12.7 | 39.5 | 6.3 | 2.0 | 4.2 | 160 | 268 | 214 |
| 6 | 4.0 | 9.0 | 6.5 | 8.7 | 16.3 | 12.5 | 8.7 | 13.3 | 11.0 | 3.7 | 4.0 | 3.8 | 162.7 | 148 | 155.3 |
|  |  |  |  |  |  |  |  |  |  |  |  |  |  |  |  |
| 7 | 41.3 | 27.3 | 34.3 | 241.7 | 61.3 | 151.5 | 112.3 | 27.7 | 70.0 | 11.0 | 1.3 | 6.2 | 170 | 82.3 | 126.2 |
| 8 | 1.0 | 103.7 | 52.3 | 3.0 | 56.3 | 29.7 | 2.7 | 23.3 | 13.0 | 1.3 | 7.7 | 4.5 | 85.3 | 127 | 106.2 |
| 9 | 3.0 | 5.0 | 4.0 | 34.0 | 153.7 | 93.8 | 15.7 | 120.0 | 67.8 | 1.7 | 8.3 | 5.0 | 29 | 122.3 | 75.7 |
| 10 | 2.3 | 74.0 | 38.2 | 7.3 | 82.3 | 44.8 | 4.0 | 50.0 | 27.0 | 0.7 | 14.0 | 7.3 | 160 | 108 | 134 |
| 11 | 16.7 | 23.0 | 19.8 | 29.7 | 75.3 | 52.5 | 25.7 | 85.3 | 55.5 | 6.3 | 4.3 | 5.3 | 80 | 119.3 | 99.7 |
| 12 | 8.0 | 66.0 | 37.0 | 14.7 | 44.0 | 29.3 | 14.0 | 81.3 | 47.7 | 4.7 | 22.0 | 13.3 | 108.7 | 93.7 | 101.2 |
| 13 | 27.3 | 21.7 | 24.5 | 132.7 | 94.7 | 113.7 | 42.0 | 45.3 | 43.7 | 6.0 | 5.7 | 5.8 | 129.7 | 150.7 | 140.2 |

| Supplementary table 2 continued | | | |  |  |  |
| --- | --- | --- | --- | --- | --- | --- |
| Case | central CD163 (/3HPF) | marginal CD163 (/3HPF) | total CD163 (/6HPF) | central CD163/CD68 ratio (/3HPF) | marginal CD163/CD68 ratio (/3HPF) | total CD163/CD68 ratio (/6HPF) |
| 1 | 69.0 | 52.0 | 60.5 | 0.36 | 0.44 | 0.39 |
| 2 | 36.3 | 169.7 | 103.0 | 0.29 | 0.70 | 0.56 |
| 3 | 72.0 | 169.0 | 120.5 | 0.57 | 1.00 | 0.81 |
| 4 | 149.7 | 175.3 | 162.5 | 1.17 | 0.66 | 0.83 |
| 5 | 92.0 | 166.7 | 129.3 | 0.58 | 0.62 | 0.60 |
| 6 | 52.3 | 71.3 | 61.8 | 0.32 | 0.48 | 0.40 |
|  |  |  |  |  |  |  |
| 7 | 169.7 | 101.7 | 135.7 | 1.00 | 1.23 | 1.08 |
| 8 | 58.0 | 125.3 | 91.7 | 0.68 | 0.99 | 0.86 |
| 9 | 26.3 | 108.7 | 67.5 | 0.91 | 0.89 | 0.89 |
| 10 | 133.0 | 163.0 | 148.0 | 0.83 | 1.51 | 1.10 |
| 11 | 55.0 | 100.3 | 77.7 | 0.69 | 0.84 | 0.80 |
| 12 | 40.0 | 82.7 | 61.3 | 0.37 | 0.88 | 0.61 |
| 13 | 148.7 | 158.7 | 153.7 | 1.15 | 1.05 | 1.10 |
